# *CNGA3* mutations in two United Arab Emirates families with achromatopsia

**Published:** 2008-07-10

**Authors:** Yachna Ahuja, Susanne Kohl, Elias I. Traboulsi

**Affiliations:** 1 Center for Genetic Eye Diseases, Cole Eye Institute, Cleveland Clinic Foundation, Cleveland, OH; 2 Molecular Genetics Laboratory, Institute for Ophthalmic Research, Centre for Ophthalmology, University of Tuebingen, Germany

## Abstract

**Purpose:**

Achromatopsia results from mutations in one of three genes: *cyclic nucleotide-gated channel, alpha-3 (CNGA3); cyclic nucleotide-gated channel, beta-3 (CNGB3); and guanine nucleotide-binding protein, alpha-transducing activity polypeptide 2 (GNAT2)*. We report the responsible mutations in two United Arab Emirates families who have this autosomal recessive disease.

**Methods:**

Clinical examinations were performed in seven patients from three nuclear families. Molecular genetic testing for common *CNGA3* and *CNGB3* mutations was undertaken using standard protocols.

**Results:**

All patients were extremely light sensitive and had reduced visual acuity and no color perception. Fundus examinations did not show any visible abnormalities. After further pedigree analysis, two of the families were found to be linked through the paternal line. Two mutations in *CNGA3* were identified: Arg283Trp and Gly397Val. Family A, the larger pedigree, had one branch in which two sisters and one brother were homozygous for the Gly397Val mutation and another branch in which a brother and sister were compound heterozygous for both aforenamed mutations. Family B, however, only had two brothers who were homozygous for the Arg283Trp mutation.

**Conclusions:**

Achromatopsia in these two United Arab Emirates families results from two different mutations in *CNGA3*. Two branches of the same pedigree had individuals with both homozygous and compound heterozygous disease, demonstrating a complex molecular pathology in this large family.

## Introduction

Achromatopsia (OMIM #216900, #26230), also known as rod monochromacy or total color blindness, is an autosomal recessive congenital cone dystrophy with an estimated incidence of less than 1 in 30,000. [1,2] The disorder presents in infancy with poor visual acuity, pendular nystagmus, severe sensitivity to light, and a complete lack of color perception. While visual acuity remains stable around 20/200 or less, nystagmus and photophobia may decrease with age. Fundus examination is usually normal with rare nonspecific macular or peripheral changes. The objective diagnosis of achromatopsia is based on electroretinography (ERG), which reveals preserved rod function and undetectable cone response [3].

Achromatopsia results from the absence of functional cones in the retina. Over the past decade there has been significant progress in unraveling the molecular genetics of this rare disorder. To date, achromatopsia has been associated with mutations in three genes that play essential roles in the cone phototransduction pathway. The first two genes discovered were *CNGA3* and *CNGB3*, which respectively encode the *α-* and *β-*subunits of the cGMP-gated channel present in cone photoreceptors [4,5]. In two large studies, pathogenic mutations found in *CNGB3* (*ACHM3* locus on chromosome 8q21) were mostly nonsense or frameshift mutations and accounted for 40%–50% of cases [6,7]. In contrast, the majority of pathogenic mutations reported in *CNGA3* (*ACHM2* locus on chromosome 2q11) have mostly been of the missense type. *CNGA3* mutations are expected to account for about 20%–30% of achromatopsia cases [8,9].

More recently, a third gene, *GNAT2*, was implicated in achromatopsia and was mapped to locus *ACHM4* on chromosome 1p13. This gene codes for the *α-*subunit of the cone photoreceptor transducin G-protein in the phototransduction pathway. Pathogenic mutations in *GNAT2* have been associated with only 2%–3% of cases in studies to date [10-12].

To our knowledge, the genetic basis of achromatopsia in United Arab Emirates has not been reported previously. We describe here the responsible mutations in two UAE families with this rare disorder.

## Methods

### *Patients* and *ophthalmologic examination*

Seven achromatopsia patients from three nuclear families were involved in this study : the patients were recruited from the Cleveland Clinic Consultation Eye Service in AbuDhabi, United Arab Emirates. There were 3 females and 4 males ranging in age from 5 to 16 years. Venous blood samples were collected from all seven patients after informed consent was obtained. IRB approval for the study was obtained from the Cleveland Clinic Foundation and the United Arab Emirates health authorities. Patients were clinically examined and followed-up in Abu Dhabi, UAE. Ophthalmologic examination included the assessment of best-corrected visual acuity, external examination, slit-lamp and funduscopic examination, and Ishihara color vision testing. Diagnosis was based on the presence of nystagmus, severe photophobia, complete lack of color discrimination, and a visual acuity of 20/100 or less. ERG was not available. In one of the younger patients, visual acuity and color vision was difficult to assess; however clinical signs and symptoms were consistent with a diagnosis of achromatopsia.

**Table 1:** Characteristics of the seven achromatopsia patients examined in this study

Family	Patient	Age	Gender	Visual acuity	Gene	Allele 1	Allele 2
A	IV:1	16	Female	0.2/0.2	CNGA3	Arg283Trp	Gly397Val
A	IV:2	14	Male	0.2/0.2	CNGA3	Arg283Trp	Gly397Val
A	III:6	10	Female	0.2/0.2	CNGA3	Gly397Val	Gly397Val
A	III:7	8	Male	0.25/0.25	CNGA3	Gly397Val	Gly397Val
A	III:8	5	Female	0.2/0.2	CNGA3	Gly397Val	Gly397Val
B	III:1	12	Male	0.05/0.05	CNGA3	Arg283Trp	Arg283Trp
B	III:3	6	Male	*	CNGA3	Arg283Trp	Arg283Trp

The asterisk indicates that the family member was not examined. A diagnosis of achromatopsia was given based on poor visual acuity with normal fundi, photophobia, nystagmus, and lack of color vision yet the presence and independent inheritance of two mutant CNGA3 alleles could not be confirmed unequivocally in either of the pedigrees as only affected patients and no parents were available for segregation analysis.

**Figure 1:**
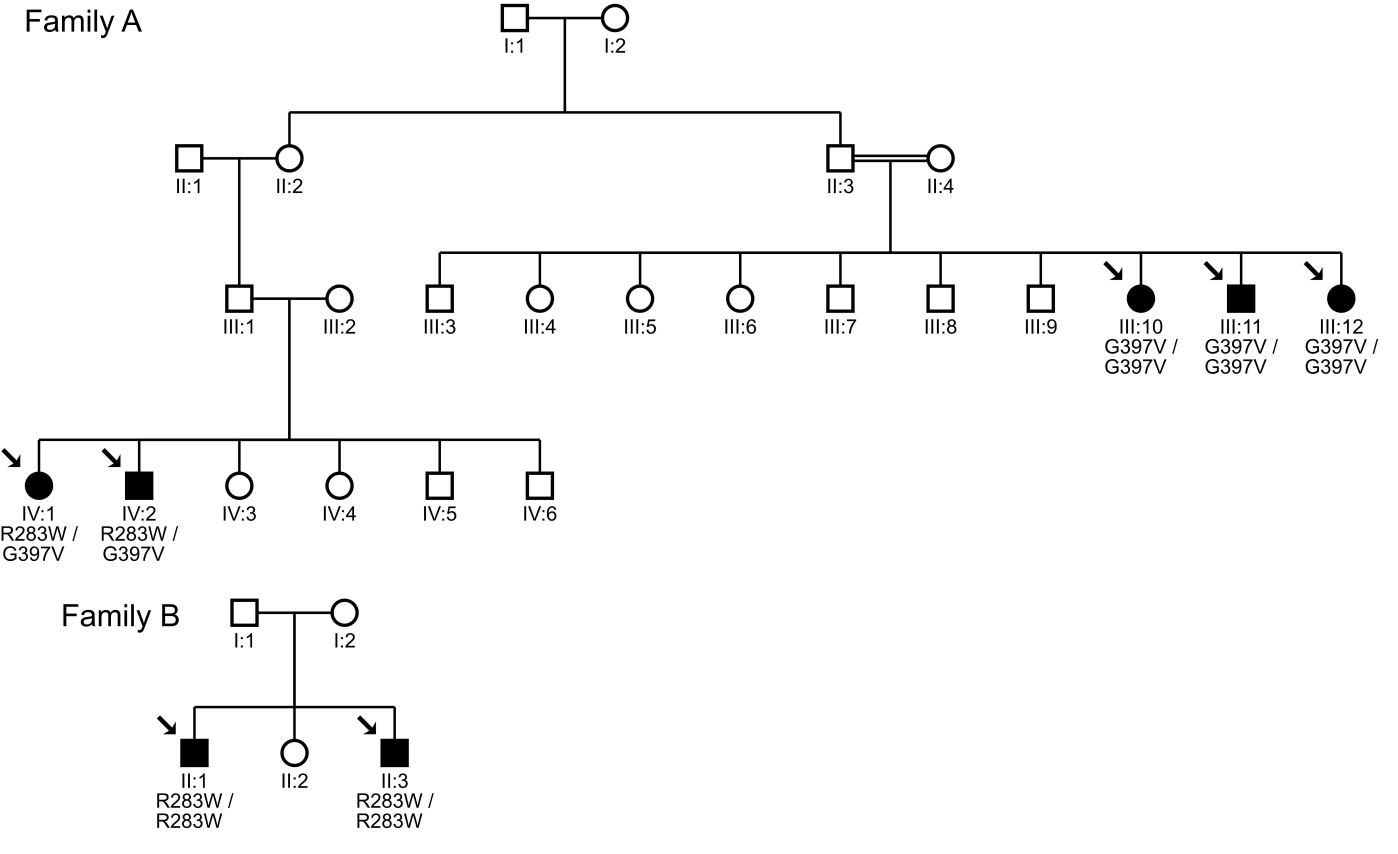
Pedigrees of family A and family B with autosomal recessive achromatopsia originating from the United Arab Emirates and carrying mutations in the CNGA3 gene. Family A has two branches carrying either the two heterozygous mutations R283W and G397V or being homozygous for the mutation G397V due to consanguinity of the parents. Patients of family B are both homozygous for the mutation R283W in the CNGA3 gene. Squares indicate males and circles females. Open symbols indicate healthy individuals, while shadings designate the affected patients. Arrows point to family members for whom DNA samples were available for genetic analysis. CNGA3 genotypes are given below each analyzed individual.

### Molecular genetic analysis

Genetic analysis was performed at the Molecular Genetic Laboratory of the University Eye Hospital, Tuebingen, Germany. DNA was extracted from venous blood samples approximately one week after the blood draw. In the intervening time the samples were refrigerated. The most common prevalent mutations in *CNGB3* were excluded by PCR/RFLP and PCR/denaturating high pressure liquid chromatography (dHPLC) analysis in the index patient from each family, as described previously [6]. All coding exons and flanking intronic sequences of *CNGA3* were amplified from genomic DNA by means of PCR [4]. PCR fragments were treated with ExoSAP (GE Healthcare, Freiburg, Germany) and subjected to direct DNA sequencing applying BigDye Terminator 1.1 chemistry (Applied Biosystems, Darmstadt, Germany). All sequences were run on an ABI 3100 DNA sequencer, and analyzed with Sequence Analysis 5.1 (Applied Biosystems) and SeqMan software (Lasergene, Madison WI). Segregation analysis was performed for all available patients within the families by direct sequencing of the relevant gene segment of exon 7 of the CNGA3 gene [6].

## Results

### Clinical findings

All patients were extremely light sensitive and had reduced visual acuity and no color perception. Pendular nystagmus was observed in all patients. Funduscopic examinations did not reveal any visible abnormalities. Patient characteristics and findings are summarized in Table 1. After further investigation of the pedigrees, it was found that two of the nuclear families were linked through the paternal line in one, shown in Figure 1A as Family A. Five children in Family A were diagnosed with achromatopsia. Family B (Figure 1B) is an independent family in which two children were affected.

### Molecular genetic findings

**Figure 2:**
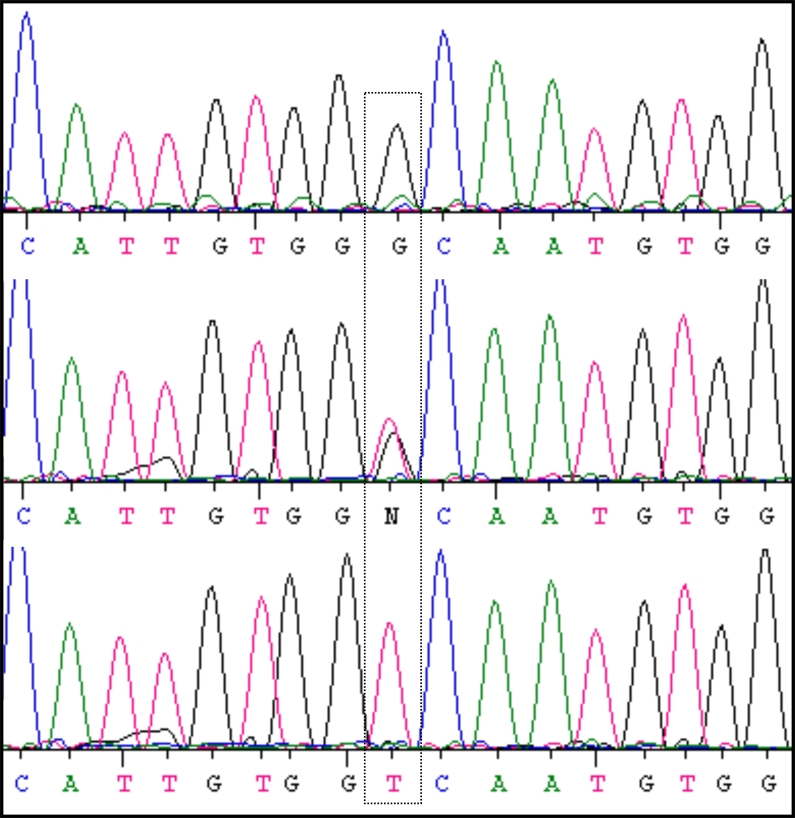
Identification of the novel *CNGA3* mutation c.1190G>T Gly397Val in Family A. Electropherogram sections of exon 7 of *CNGA3*. Selected heterozygous or homozygous mutant sequences (middle and bottom rows) compared with the respective wild-type sequence (top row). Dotted framed encloses the nucleotide altered by the mutation.

We were able to identify either homozygous or two heterozygous mutations in all seven patients from the two families, concordant with an autosomal recessive mode of inheritance (see Table 1). The two causative mutations, c.847C>T p.Arg283Trp and c.1190G>T p.Gly397Val (Figure 2), were both found in *CNGA3* (GenBank NM_001079878). In Family A, the pedigree consisted of one branch in which two sisters and one brother were homozygous for the Gly397Val mutation, and another branch in which a brother and his sister were both compound heterozygous for Gly397Val and Arg283Trp. In Family B, two brothers were homozygous for the Arg283Trp mutation.

The mutation Arg283Trp is a missense mutation and has already been reported in the literature [4,8,13,14]. The mutation Gly397Val is to date unique to the patients of Family A and has not been previously reported. It is a missense mutation substituting a highly conserved amino acid residue in transmembrane domain S6 of the *α-*subunit of the cone cyclic-GMP gated channel.

## Discussion

Achromatopsia-associated genetic mutations have been studied in centers around the world, though mainly in Northern European countries and in Japan. Mutations in *CNGA3*, *CNGB3*, and *GNAT2* have been associated with the majority of studied cases of achromatopsia. To date, the distribution of genetic mutations in UAE or other Arab achromatopsia patients has not been assessed.

We have identified pathogenic mutations in *CNGA3* in two independent UAE achromatopsia families. As is true for the majority of known *CNGA3* mutations, the two mutations we identified are missense mutations. Of the two mutations, Gly397Val has not been reported previously and may be unique to UAE patients. We identified this mutation in a family with a complex pedigree in which one branch had three individuals who were homozygous for the Gly397Val mutation, and the other branch had two individuals who were compound heterozygous for Gly397Val and a second *CNGA3* mutation, Arg283Trp.

The Arg283Trp mutation, which was also identified in two homozygous individuals in the second family of our study, has been previously reported in patients from other parts of the world. Wissinger et al., [8] in a multinational study of 58 independent patients with *CNGA3* mutations, found the most prevalent mutation was Arg283Trp. Together with three other recurrent *CNGA3* mutant alleles, this mutation accounted for nearly 42% of the total identified mutant alleles in this large study. Even though there is a high occurrence of Arg283Trp in two different geographic locations—in northern Europe and in northern Italy—further segregation analysis and haplotype reconstruction of the Arg283Trp segregating alleles in the study suggested that all observed Arg283Trp chromosomes share a common origin. Independent studies of Hungarian and Swedish patients have also reported the Arg283Trp mutation [13,14] (The Swedish patients are included in the Wissinger study [8].). To date, however, the Arg283Trp mutation has not been reported in Japanese patients or in patients in the United Kingdom [7,9,15]. Though our study is of a much smaller scale than these studies, it is notable that the Arg283Trp mutation was independently present in both UAE families.

We noted that subject III:6 from Family B, who is homozygous for the aforementioned Arg283Trp mutation, appears to also have the worst visual acuity compared to the other subjects. The severity of the disease in this patient is within the spectrum expected in this disorder, but yet worse than others in his age group and ethnic background. There have been functional analyses in our laboratory and by other groups on this mutation by heterologous in vitro expression of mutant CNGA3 channels carrying this mutation in HEK293 cells. It was shown to be nonfunctional (functional null mutation), being compatible with a phenotype of complete achromatopsia [16].

In the multinational study by Wissinger et al., [8] it was estimated that *CNGA3* mutations were associated with only about 25% of achromatopsia cases. A more recent study in United Kingdom achromatopsia families showed that *CNGA3* mutations accounted for up to 40% of the achromatopsia cases in the 22 families studied [9]. In contrast, our study showed that the cases of achromatopsia in the two UAE families we examined were associated with *CNGA3* mutations. The frequency of *CNGA3* mutations in UAE achromatopsia patients in this relatively small population (less than 1 million) remains to be determined.

For several decades now, the estimated worldwide prevalence of achromatopsia is quoted as 1 in 30,000. [1,2] Certain isolated populations with a considerably higher incidence than this figure have, however, been discovered and studied extensively. For example, the disease incidence in the Pingelapese islanders of Micronesia was reported as 1:20 with a prevalence of a single specific *CNGB3* founder mutation due to gene drift and inbreeding [17-19]. More recently, Rojas et al. [20,21] reported that the incidence of achromatopsia in a rural isolate in central Chile is 1:60 and is associated with unique *CNGB3* mutations. The present study is not an epidemiologic study and does not allow the determination of the contribution of achromatopsia to the burden of childhood blindness in this country. However, there is a high rate of consanguinity in the UAE and according to a 1997 demographic study, the rate has increased from 39% to 50.5% in one generation. This may consequently play a large role in the high frequencies of recessive genetic disorders in this population [22-24]. Furthermore, UAE nuclear families are generally large with several children in any particular case; we have often dealt with families with up to 12 children [25].

Population-wide screening for *CNGA3* mutations, in addition to genetic counseling in UAE families and subpopulations affected by achromatopsia, could be undertaken to determine carrier status and to provide informed risk for individual families.
